# Impact of age and cardiovascular risk factors on the incidence of adverse events in patients with rheumatoid arthritis treated with Janus Kinase inhibitors: data from a real-life multicentric cohort

**DOI:** 10.1007/s10238-024-01325-z

**Published:** 2024-03-30

**Authors:** Stefano Gentileschi, Carla Gaggiano, Arianna Damiani, Carmela Coccia, Pamela Bernardini, Massimiliano Cazzato, Francesco D’Alessandro, Giulia Vallifuoco, Riccardo Terribili, Marco Bardelli, Caterina Baldi, Luca Cantarini, Marta Mosca, Bruno Frediani, Serena Guiducci

**Affiliations:** 1 Rheumatology Unit, Department of Medicine, Surgery and Neurosciences, Azienda Ospedaliero-Univeristaria Senese, University of Siena, Siena, Italy; 2 Division of Rheumatology, Department of Experimental and Clinical Medicine, University of Florence, Florence, Italy; 3 Rheumatology Unit, Department of Clinical and Experimental Medicine, University of Pisa, Pisa, Italy

**Keywords:** Rheumatoid arthritis, JAK inhibitors, Safety, Cardiovascular risk factors

## Abstract

Inhibiting Janus Kinases (JAK) is a crucial therapeutic strategy in rheumatoid arthritis (RA). However, the use of JAK inhibitors has recently raised serious safety concerns. The study aims to evaluate the safety profile of JAKi in patients with RA and identify potential risk factors (RFs) for adverse events (AEs). Data of RA patients treated with JAKi in three Italian centers from January 2017 to December 2022 were retrospectively analyzed. 182 subjects (F:117, 64.3%) underwent 193 treatment courses. 78.6% had at least one RF, including age ≥ 65 years, obesity, smoking habit, hypertension, dyslipidemia, hyperuricemia, diabetes, previous VTE or cancer, and severe mobility impairment. We identified 70 AEs (28/100 patients/year), among which 15 were serious (6/100 patients/year). A high disease activity was associated with AEs occurrence (*p* = 0.03 for CDAI at T0 and T6; *p* = 0.04 for SDAI at T0 and T6; *p* = 0.01 and *p* = 0.04 for DAS28ESR at T6 and T12, respectively). No significant differences in AEs occurrence were observed after stratification by JAKi molecules (*p* = 0.44), age groups (*p* = 0.08) nor presence of RFs (*p* > 0.05 for all of them). Neither the presence of any RFs, nor the cumulative number of RFs shown by the patient, nor age ≥ 65 did predict AEs occurrence. Although limited by the small sample size and the limited number of cardiovascular events, our data do not support the correlation between cardiovascular RFs—including age—and a higher incidence of AEs during JAKi therapy. The role of uncontrolled disease activity in AEs occurrence should by emphasized.

## Introduction

Rheumatoid arthritis (RA) is a chronic, immune-mediated condition affecting multiple systems, which can result in progressive joint damage, functional impairment, and relevant comorbidities. Timely diagnosis and prompt treatment are essential to alleviate symptoms, prevent chronic complications, and reduce the overall impact of comorbidities associated with the disease [1]. To date, cardiovascular (CV) disease represents the leading cause of mortality in individuals with RA [2], with a higher risk observed in RA patients compared to the general population [3].

Over the last decades, the treatment options for RA have significantly expanded and diversified, including biological disease-modifying drugs (bDMARDs) and Janus Kinase inhibitors (JAKi), capable of substantially improving disease control, quality of life and long-term prognosis for these patients. Also, the increasing familiarity with the different molecules and mechanisms of action is making personalized therapy an increasingly attainable goal. Even if evidence about the existence of biomarkers able to predict patients’ response to therapy is still lacking, data are emerging about the utility of patients’ profiling to guide more targeted treatment choices. As examples, the need of a monotherapy because of contraindication to conventional DMARDs (cDMARDs) could drive to prefer tocilizumab, sarilumab or JAKi; pregnancy desire could move the decision onto certolizumab Pegol; anti-citrullinated protein antibodies (ACPAs) and or rheumatoid factor positivity could lead to choose rituximab or abatacept [4, 5]. Moreover, recent pharmacogenomic studies are assessing the role of specific genes in predicting response to different molecules [6] and research on synovial biopsy is being conducted to identify histopathological biomarkers [7, 8]. Nevertheless, no universal consensus is available on this topic and no definite evidence emerged from the systematic review of the available literature [9]. Indeed, safety remains mandatorily the leading guide in treatment choice [10].

The use of JAKi has recently raised concerns due to the potential occurrence of serious (S) adverse events (AEs), including CV and venous thromboembolic events (VTE), as emerged from the ORAL surveillance study and further observational studies on RA patients treated with tofacitinib (TOFA) and baricitinib (BAR) [11–13]. Based on these emerging data, the European Medicines Agency and the Pharmacovigilance Risk Assessment Committee have taken steps to minimize the risks of serious adverse events. Consequently, the 2022 updated EULAR recommendations on RA underscore the importance of considering age, smoking history, CV risk factors, malignancy, and prior VTE when prescribing JAKi [14]. The influx of these novel guidelines pertaining to the utilization of JAKi has undeniably prompted a change in the decision-making process among rheumatologists selecting appropriate treatments for RA patients [15]. This more cautious approach could potentially lead to a decrease in the utilization of JAK inhibitors, despite their numerous ad-vantages in terms of administration route, speed of action, and also the evidence showcasing their superior efficacy over tumor necrosis factor (TNF) inhibitors (TNFi) as highlighted in different studies [16–18].

This study aims to evaluate the safety profile of JAKi in a real-life setting by assessing the incidence of AEs and SAEs in patients with RA and exploring their potential associations with risk factors (RFs), in particular with those highlighted by the ORAL surveillance study and included in latest EULAR recommendations.

## Materials and methods

Data of patients affected by RA and treated with JAKi in three Italian referral centers (Siena University Hospital, Firenze Careggi University Hospital, and Pisa University Hospital) from January 2017 to December 2022 were retrospectively collected. Demographic, clinical and therapeutic data were collected through a standardized data sheet at the start of the JAKi therapy (T0), at 6-, 12-, 18, 24 months (T6, − 12, − 18 and − 24) and at the last follow-up visit.

Patients were classified according to the 2010 ACR/EULAR classification criteria for RA [19]. Following the directive 2001/20/EC of the European Parliament and of the Council of 4 April 2001, AEs were defined as any untoward medical occurrences associated with the use of a JAKi molecule, whether considered drug-related or not. AEs were considered “serious” if, in the view of the investigator, they resulted in any of the following outcomes: death, a life-threatening condition, inpatient hospitalization or prolongation of existing hospitalization, a persistent or substantial disruption of the ability to conduct normal life functions, or a congenital anomaly/birth defect.

The incidence rates of AEs and SAEs were computed by determining the ratio of the number of events to the total time at risk, based on the duration of JAKi exposure for each patient. Safety data were analyzed according to the presence or absence of the following risk factors: age ≥ 65 years at the start of the therapy, body mass index (BMI) ≥ 30 kg/m2, smoking habit, hypertension, hypercholesterolemia, high density lipoprotein (HDL) < 40 mg/dl, hypertriglyceridemia, hyperuricemia, diabetes, previous VTE, history of cancer, and severe mobility impairment. In addition, safety data were analyzed according to the patient’s disease activity level at the different timepoints, measured through the Clinical Disease Activity Index (CDAI), the Simple Disease Activity Index (SDAI) and the Disease Activity Score 28 based on erythrocyte sedimentation rate value (DAS28 ESR).

Statistical analysis was performed by using JASP open-source statistics package version 0.16.3. Descriptive statistics included sample sizes, mean and standard deviation or median and interquartile range (IQR). The Shapiro–Wilk test was used to assess normality distribution of data. Associations between categorical variables were analyzed using contingency tables with the Chi-Square test with Yates' continuity correction. Statistical difference between the medians of two independent groups was determined by the Mann–Whitney test. Dichotomic outcomes were predicted by logistic regression analysis. The threshold for statistical significance was set to *p* < 0.05 and all *p*-values were two-sided.

The study was conducted according to the guidelines of the declaration of Helsinki and approved by the local ethics committee (Rhelabus 22,271). Written informed consent was obtained from all subjects involved in the study.

## Results

We enrolled 182 patients (F:117, 64.3%) undergoing a total of 193 treatment courses with JAKi (median follow-up of 12 [IQR 9] months, range 1–60). The mean ± SD age of the patients was 62.2 ± 11.7 years. Patients’ demographic and clinical information are showed in Table 1. TOFA was used in 35 cases (18.1%), BAR in 84 (43.5%), upadacitinib (UPA) in 50 (25.9%), and filgotinib (FIL) in 24 (12.4%).

**Table 1:** Demographic, clinical, and therapeutic data of the study cohort

*Patients n = 182*	
Age, years (mean ± SD)	62.3 ± 11.8
Female	117 (64.3%)
RF +	136 (74.7%)
ACPA +	121 (66.5%)
Pulmonary involvement	10 (5.5%)
Ocular involvement	49 (26.9%)
Osteoporosis	36 (19.8%)
Fibromyalgia	15 (8.2%)
*JAKi treatment courses n = 193*	
JAKi treatment duration, months (median [IQR])	12 [9]
Previous csDMARDs therapy	147 (76.2%)
bDMARDs naïve	61 (31.6%)
Concomitant csDMARDs therapy	56 (29.0%)
Concomitant Cs therapy	148 (76.7%)
Concomitant Cs highest posology, mg/day (median [IQR])	7.5 [7.5]
DAS28 ESR at T0 (mean ± SD)	4.1 ± 1.3
CDAI at T0 (mean ± SD)	21.9 ± 10.9
SDAI at T0 (mean ± SD)	22.7 ± 11.3

*ACPA* anti-citrullinated protein antibodies; *bDMARDs* biologic synthetic disease modifying anti-rheumatic drugs; *CDAI* clinical disease activity index; *Cs* corticosteroids; *csDMARDs* conventional synthetic disease modifying anti-rheumatic drugs; *DAS28 ESR* disease activity score 28 based on erythrocyte sedimentation rate; *IQR* interquartile range; *JAKi* Janus Kinase inhibitors; *RF* rheumatoid factor; *SD* standard deviation; *SDAI* simple disease activity index; *T0* baseline assessment of JAKi therapy

### Risk factors

**Fig. 1:**
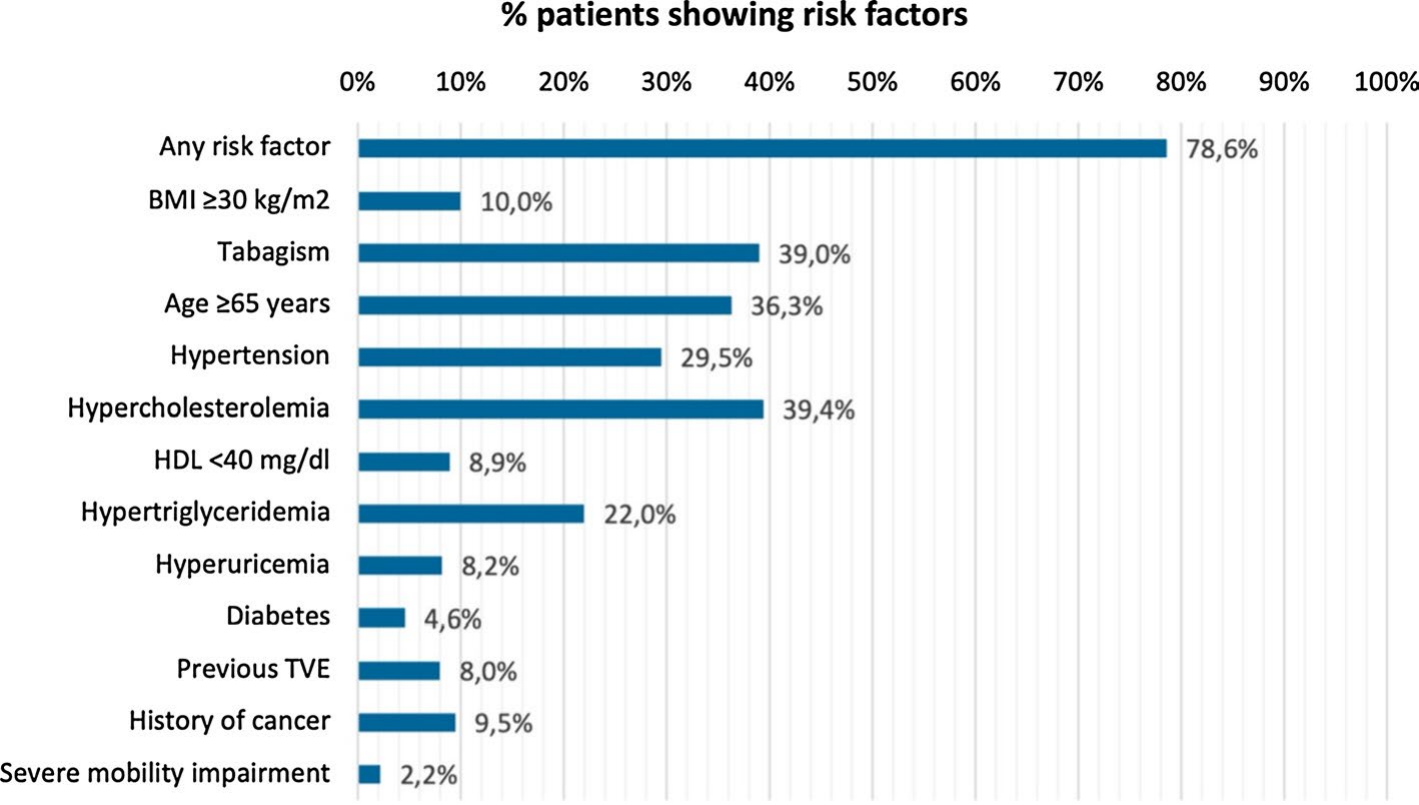
Percentages of patients showing risk factors for possible adverse events during JAKi therapy. *BMI* body mass index; *HDL* high density lipoproteins; *TVE* venous thromboembolic events

**Fig. 2:**
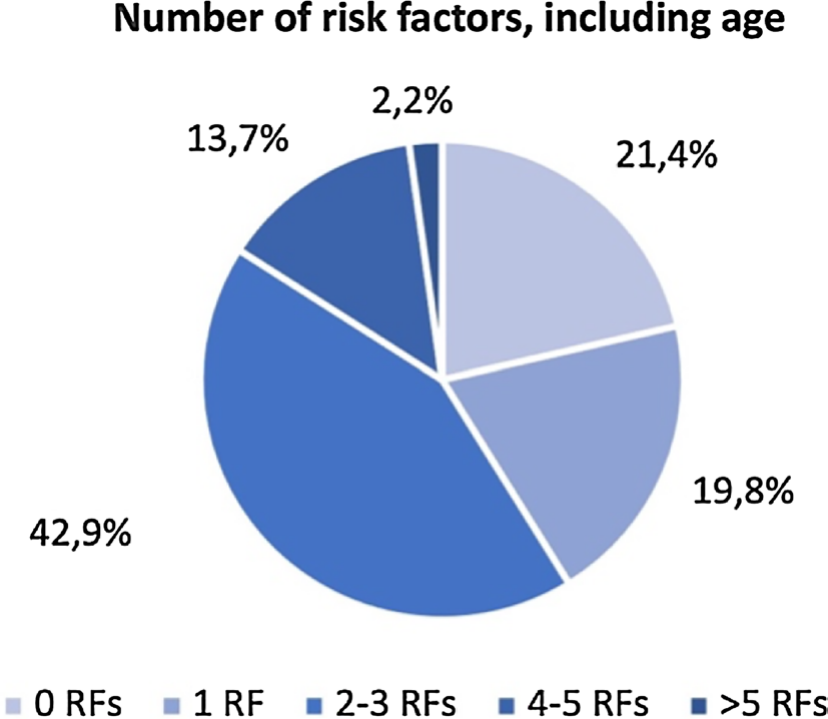
Percentage of patients showing 0, 1 or more than 1 risk factor(s), including age ≥ 65 years, in our cohort. *RFs* risk factors

Among the patients, 143 (78.6%) showed at least one RF at the start of JAKi, including age ≥ 65 years, BMI ≥ 30 kg/m2, smoking habit, hypertension, hypercholesterolemia, HDL < 40 mg/dl, hypertriglyceridemia, hyperuricemia, diabetes, previous VTE, history of cancer, and severe mobility impairment. Figures 1 and 2 show the frequency of RFs in our cohort.

There were no statistically significant differences in the median follow-up duration according to the presence of RFs or age ≥ 65 years (*p* = 0.53 and 0.73, respectively).

### Adverse events

We identified 70 AEs, among which 15 were considered severe (SAEs): ocular toxoplasmosis in 1 case, septic arthritis in 1, popliteal vein thrombosis in 1, pulmonary carcinoma in 1, pneumonitis in 4, interstitial pneumonitis in 2, myocardial infarction in 1, myocardial infarction MINOCA type in 1, Herpes Simplex virus (HSV) keratitis in 1, bacterial keratitis in 1, and 1 hospital admission for unspecified reason. The incidence rates of AEs and SAEs were 28 and 6 per 100 patients/year, respectively.

**Fig. 3:**
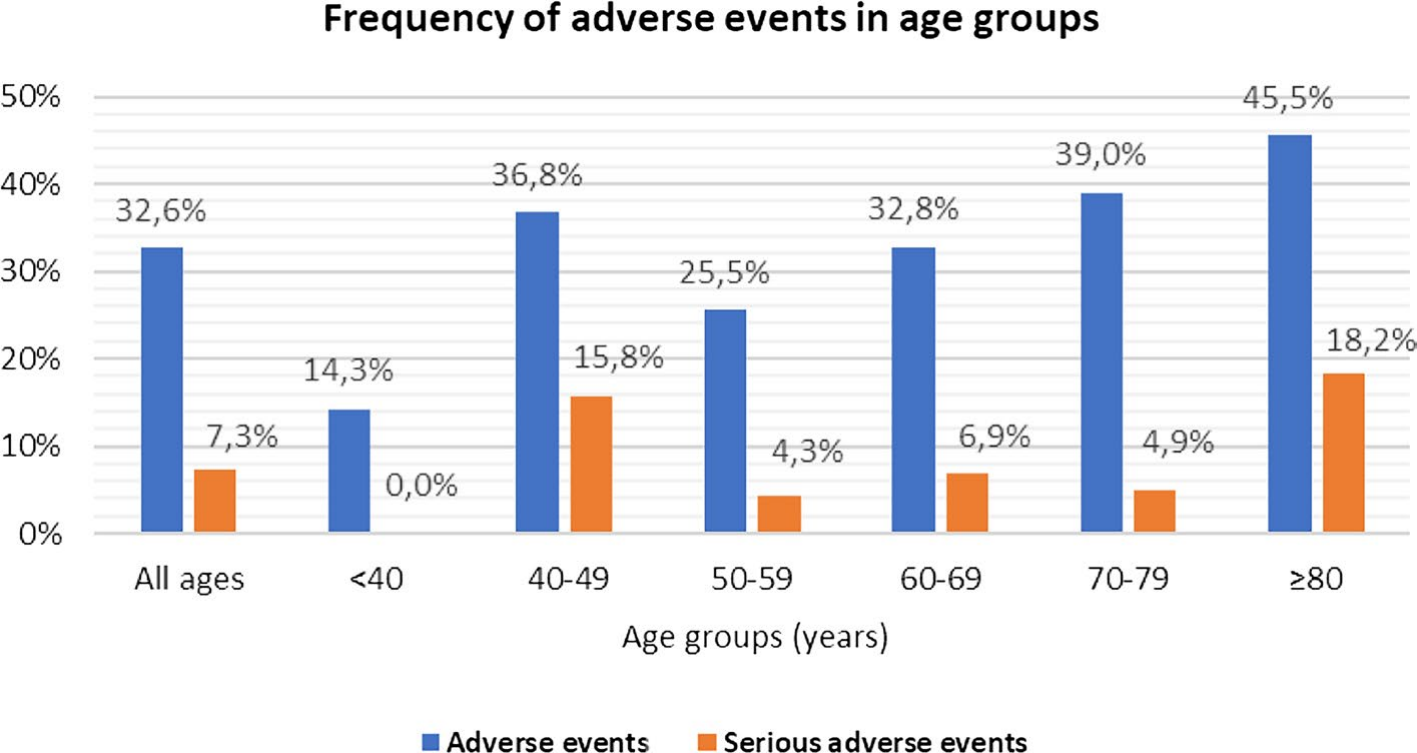
Frequency of adverse events in different age groups

More in detail, the incidence rate of infections, serious infections and herpetic reactivations were 21.6, 3.6 and 8.4 per 100 patients/year, respectively. Adverse events led to treatment suspension in 24 cases (38.1%). Three thromboembolic events (myocardial infarction, n = 2; deep venous thrombosis, n = 1, 1.2 per 100 patients/year) and 21 herpesvirus reactivations (Varicella-Zoster virus [VZV] or HSV) were reported. Further details of AEs reported are displayed in Table 2. Figure 3 shows the frequency of adverse events in different age groups in our cohort.

**Table 2:** Number and percentages of treatment courses affected by adverse events in the whole cohort and stratifying patients according to the age and presence of risk factors

	All treatment courses (n = 193)	Age ≥ 65 years (n = 69)	Age < 65 years (n = 124)	RFs (excluding age) (n = 139)	No RFs (excluding age) (n = 54)
AEs	63 (32.6%)	28 (40.6%)	35 (28.2%)	51 (36.7%)	12 (22.2%)
SAEs	14 (7.3%)	7 (10.1%)	7 (5.6%)	10 (7.2%)	4 (7.4%)
EA-related drug discontinuation	24 (12.4%)	12 (17.4%)	12 (9.7%)	22 (14.3%)	2 (5.1%)
EAs thromboembolic	3 (1.6%)	3 (4.3%)	0 (0%)	2 (1.4%)	1 (1.9%)
EAs infectious Respiratory Urinary Cutaneous Herpetic Others	46 (23.8%) 16 (8.3%) 8 (4.1%) 4 (2.1%) 21 (10.9%) 7 (3.6%)	18 (26.1%) 5 (7.2%) 4 (5.8%) 2 (2.9%) 9 (13.0%) 2 (2.9%)	28 (22.6%) 11 (8.9%) 4 (3.2%) 2 (1.6%) 12 (9.7%) 5 (4.0%)	37 (26.6%) 12 (8.6%) 7 (5.0%) 3 (2.2%) 20 (14.4%) 5 (3.6%)	9 (16.7%) 4 (7.4%) 1 (1.9%) 1 (1.9%) 1 (1.9%) 2 (3.7%)
EAs neoplastic	1 (0.5%)	1 (1.4%)	0 (0%)	1 (0.7%)	0 (0%)
EAs others	12 (6.2%)	5 (7.2%)	7 (5.7%)	9 (6.5%)	3 (5.6%)

*AEs* adverse events; *RFs* risk factors; *SAEs* severe adverse events

### Association of AEs with possible risk factors

There were no statistically significant differences when comparing patients aged < 65 and ≥ 65 years concerning the occurrence of AEs (*p* = 0.08) or treatment suspension due to AEs (*p* = 0.17). No differences were found between patients with or without AEs according to the presence of BMI ≥ 30 kg/m2 (*p* = 0.16), smoking habit (*p* = 0.13), hypertension (*p* = 0.08), hypercholesterolemia (*p* = 0.21), HDL < 40 mg/dl (*p* = 0.54), hypertriglyceridemia (*p* = 0.2), hyperuricemia (*p* = 0.96), diabetes (*p* = 0.09), previous VTE (*p* = 0.37), history of cancer (*p* = 0.28), and severe mobility impairment (*p* = 0.85). The presence of any of the RFs was not associated with treatment discontinuation due to AEs (*p* = 1.00).

Stratifying the treatment regimens based on the JAKi used, no significant differences were observed in the frequency of AEs (*p* = 0.44), including herpetic virus reactivation (*p* = 0.25), and in the frequency of AE-related drug discontinuation (*p* = 0.28).

**Fig. 4:**
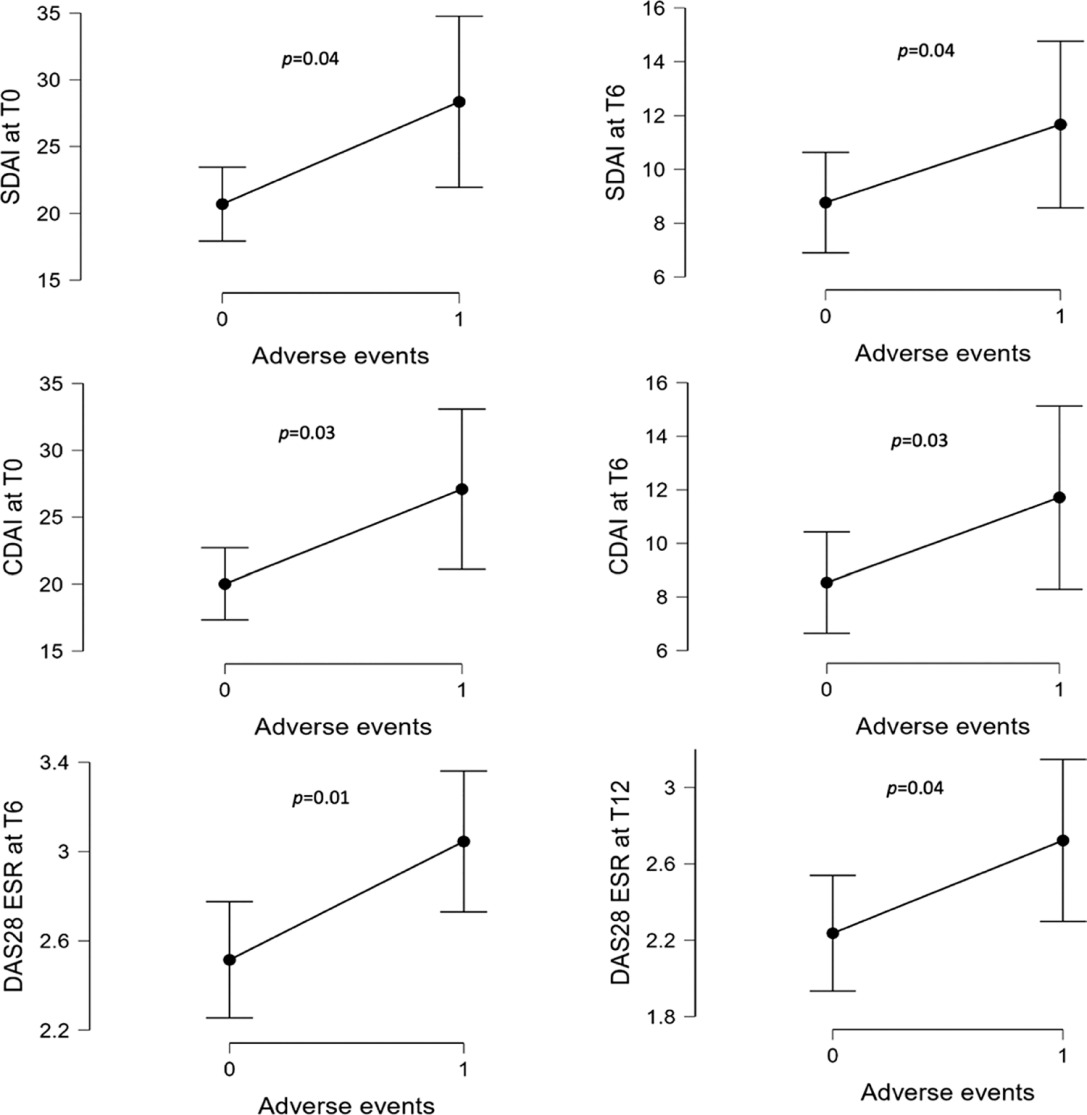
Distribution of the disease activity indexes at different timepoints (baseline, 6 and 12 months) in patients experiencing and not experiencing adverse events during JAKi therapy. *CDAI* clinical disease activity index; *DAS28 ESR* disease activity score 28 based on erythrocyte sedimentation rate; *JAKi* Janus Kinase inhibitors; *SDAI* simple disease activity index

Median disease activity indexes, namely CDAI at T0 (*p* = 0.03) and T6 (*p* = 0.03), SDAI at T0 (*p* = 0.04) and T6 (*p* = 0.04); DAS28ESR at T6 (*p* = 0.01) and T12 (*p* = 0.04), were significantly higher in patients experiencing AEs during treatment than in subjects without AEs (Fig. 4).

According to the logistic regression analysis, neither the presence of any RFs nor the age ≥ 65 years nor the cumulative number of RFs shown by the patient may significantly predict the occurrence of AEs.

## Discussion

As of today, the JAK inhibitors approved in Europe for the treatment of RA are tofacitinib, baricitinib, upadacitinib, and filgotinib. These molecules have demonstrated superiority over placebo in treating active rheumatoid arthritis unresponsive to conventional synthetic DMARDs [20]. The JAK-signal transducer and activator of transcription (STAT) pathway is a major downstream intracellular signaling system that plays a crucial role in orchestrating immune responses and controlling hematopoiesis and inflammation. JAKi, with varying selectivity for JAK1, JAK2, JAK3, and Tyk2, have then the role of disrupting proinflammatory cytokine cascades in rheumatic diseases [21]. With the release of the new EULAR guidelines concerning the utilization of JAK inhibitors for RA treatment [14], there is potential for a defensive medicine scenario. This could result in patients not receiving the most appropriate therapeutic option due to the presence of RFs, effectively hindering the achievement of precision medicine. Therefore, there is an increasing need for safety data from both randomized controlled trials (RCTs) and real-world cohorts to better understand the role of individual RFs in the occurrence of AEs during JAKi treatment, especially considering that comparative data from registries and real-world settings regarding SAEs, and neo-plasms, currently conflict with those from the ORAL surveillance study [22–26]. In a “real world” multi-database study by Farzin et al. on RA patients, no evidences emerged for increased risk on CV outcomes with TOFA, when compared to TNFi [22]. Nevertheless, the authors of the paper highlight a higher, though statistically non-significant, risk of CV events associated with TOFA, as opposed to individuals treated with TNFi, among those with existing CV RFs or a history of prior CV events [22]. In a Taiwanese registry of patients with RA, a total of 3179 subjects were examined, including 2357 treated with TNFi and 822 treated with JAKi [23]. Regarding coronary disease, the incidence was 0.48 and 0.45 per 100 patients/year for patients treated with JAKi and TNFi, respectively (*p* = 0.94). The incidence of stroke was 0.33 and 0.46 per 100/patient years for subjects treated with JAKi and TNFi, respectively (*p* = 0.55), while the incidence of deep venous thrombosis was 0.26 and 0.44 for patients treated with JAKi and TNFi, respectively (*p* = 0.3). Finally, the incidence for malignancies was 0.39 in the JAKi group and 0.35 in the TNFi group (*p* = 0.83). Data from this paper showed similar safety outcomes, risk of AE and mortality in the JAKi group compared with the TNFi group [23]. Accordingly, data from the CORRONA registry showed similar AE (serious infectious events, major adverse CV events, malignancy, death and VTE) rates between RA patients treated with JAKi and various bDMARDs, with the exception of VZV reactivations, which had a significantly higher rate among the JAKi group (HR 2.32; 95% CI) [24]. A Korean study by Cho et al. on 346 RA patients showed a higher frequency of AEs reported among patients treated with JAKi, but the difference was not statistically significant (75 of 196 patients [38.3%] vs. 43 of 150 patients [28.73%], *p* = 0.105) [25]. Regarding SAEs, there was no intergroup difference in the frequency between subjects treated with JAKi and bDMARDs (4.6% vs. 4.0%, respectively, *p* = 0.789) [25]. Finally, a retrospective analysis of the Hong Kong Biologics Registry (2471 RA patients, 551 treated with JAKi and 1920 treated with TNFi) did not show an increase of major CV events (incidence 1.34 [JAKi] vs 0.75 [TNFi] per 100 patient-years; *p* = 0.22) or malignancies (0.81 [JAKi] vs 0.85 [TNFi] per 100 patient-years; *p* = 0.25) in patients treated with JAKi when compared to TNFi users [26]. Contrarily, in the same cohort there was a higher incidence for non-serious infections (16.3 vs 9.9 per 100 patient-years; *p* = 0.02) and HZV reactivation (3.49 vs 0.94 per 100 patient-years; *p* < 0.001) in the JAKi group when compared to TNFi users [26].

In our cohort, most patients (78.6%) presented at least one RF and more than half subjects presented 2 or more RFs, with hypercholesterolemia, smoking habit, age ≥ 65 years and hypertension being the most frequently reported. Regarding AEs, we reported an incidence rate of 28 AEs and 6 SAEs per 100 patients/year, respectively, while the incidence rates of infections, serious infections, and herpesvirus reactivations were 21.6, 3.6, and 8.4 per 100 patients/year, respectively. Moreover, in our cohort, around 12% of patients discontinued the treatment for safety reasons, in line with previous data on BAR from the real-world setting showing a 9.5% frequency of drug discontinuation due to AEs [27]. Concerning serious infections, the incidence rate computed from our data is consistent with what is reported in RCTs [28–30], while the incidence rate of herpesvirus reactivation is higher in our cohort than in controlled studies [30, 31]. The last finding could be explained by the real-world setting in which patients do not go through a selection process that often excludes subjects with comorbidities and/or older age. Furthermore, it should be mentioned that, in our cohort, only one patient received anti-VZV vaccination at the start of the therapy, as the recombinant peptide vaccine has only recently become available in the Italian market.

As for thromboembolic events, we observed one case of myocardial infarction in a patient treated with UPA, one case of myocardial infarction MINOCA type in a patient treated with TOFA, and one deep venous thrombotic event in a patient treated with BAR. All patients who experienced a CV event or VTE were over 65 years of age. We calculated a 1.2/100 patients/year incidence rate for VTE, which is consistent with the recently published data from Hong Kong biologics registry, where a 1.34 rate was calculated for CV, cerebrovascular and peripheral vascular events [26]. We could not demonstrate a clear association between the presence of RFs and the occurrence of CV and/or thromboembolic AEs during JAKi therapy. However, given the low number of such events in this cohort, we are unable to draw firm conclusions.

Overall, we did not find a correlation between the frequency of AEs and age ≥ 65 years per se, since in our cohort there were two peaks, one in subjects older than 70 and the other in those in their forties. On the other hand, despite lacking statistical significance, the majority of subjects who experienced AEs had at least one of the RFs studied, although this association had no impact on the frequency of treatment discontinuation. This finding sounds reasonable since most CV RFs—both in RA and the general population—also increase the risk of infection (smoking habit, diabetes and reduced mobility to cite a few). In our cohort, disease activity measured by SDAI, CDAI, and DAS28-ESR was significantly higher both at the baseline and during treatment in subjects who developed AEs than those who didn’t. It is well-known from the literature and the clinical practice that a higher disease activity is associated with a higher incidence of AEs in patients with RA. In a large US national cohort study investigating the risk of serious infections in patients with RA compared to those with non-inflammatory rheumatic diseases, the risk of all serious infections, particularly bacterial, respiratory, sepsis, skin, bone and joint infections were significantly increased in patients with RA, and it was higher in those with higher disease activity [32].

Finally, we did not observe differences in the frequency of AEs, including VZV reactivation, when stratifying the patients by the different JAKi molecules used. This last finding could be influenced by the limited sample size as well as the inhomogeneous distribution of the different molecules in our cohort. With this regard, further studies directly comparing different JAKi would be useful to understand whether differences in selectivity for different JAK could influence the tendency to develop VZV reactivation, which stands out as a class-specific AE.

This study has some limitations, such as the absence of a control group to compare safety data in RA patients treated with different DMARD classes, including bDMARDs. This could have provided a more comprehensive pool of information to utilize in the clinical setting, assisting in the selection of the most suitable, safe and effective therapies from a broad array of options available for each patient. Moreover, the small sample size and the limited observation period may have hindered the detection of less common AEs such as cancer, CV or TVE. While this provides reassurance in some respects, it also prevented us from gaining a more profound understanding of one of the major events of interest.

In conclusion, our data did not reveal any direct correlations between the presence of the examined risk factors, including age ≥ 65 years, and a higher frequency of adverse events (AEs). Conversely, a high disease activity seemed to be related with the occurrence of AEs. Therefore, while using a JAKi, it is crucial to conduct a comprehensive patient evaluation that shouldn’t be solely driven by the presence of RFs but also by the pivotal targets of suppressing systemic inflammation and effectively controlling disease activity.

## Data Availability

The datasets generated during and/or analyzed during the current study are available from the corresponding author on reasonable request.

## References

[1] Smolen JS, Aletaha D, Barton A, et al. Rheumatoid arthritis. Nat Rev Dis Primers. 2018;4:18001. 10.1038/nrdp.2018.1. (**PMID: 29417936**). 29417936 10.1038/nrdp.2018.1

[2] Young A, Koduri G, Batley M, et al. Mortality in rheumatoid arthritis. Increased in the early course of disease, in ischaemic heart disease and in pulmonary fibrosis. Rheumatology (Oxford). 2007;46:350–722. 10.1093/rheumatology/kel253. 16908509 10.1093/rheumatology/kel253

[3] Aviña-Zubieta JA, Thomas J, Sadatsafavi M, et al. Risk of incident cardiovascular events in patients with rheumatoid arthritis: a meta-analysis of observational studies. Ann Rheum Dis. 2012;71:1524–9. 10.1136/annrheumdis-2011-200726. 22425941 10.1136/annrheumdis-2011-200726

[4] Cantini F, Goletti D, Benucci M, et al. Tailored first-line biologic and targeted synthetic disease modifying anti-rheumatic drugs therapy in patients with rheumatoid arthritis: 2021 updated ITABIO statements. Expert Opin Drug Saf. 2022;21(5):613–23. 10.1080/14740338.2022.2020247. 34937466 10.1080/14740338.2022.2020247

[5] Martin-Mola E, Balsa A, García-Vicuna R, et al. Anti-citrullinated peptide antibodies and their value for predicting responses to biologic agents: a review. Rheumatol Int. 2016;36(8):1043–63. 10.1007/s00296-016-3506-3. 27271502 10.1007/s00296-016-3506-3

[6] Lim SH, Kim K, Choi CI. Pharmacogenomics of monoclonal antibodies for the treatment of rheumatoid arthritis. J Pers Med. 2022;12(8):1265. 10.3390/jpm12081265. 36013214 10.3390/jpm12081265 PMC9410311

[7] Rivellese F, Surace AEA, Goldmann K, et al. Rituximab versus tocilizumab in rheumatoid arthritis: synovial biopsy-based biomarker analysis of the phase 4 R4RA randomized trial. Nat Med. 2022;28(6):1256–68. 10.1038/s41591-022-01789-0. 35589854 10.1038/s41591-022-01789-0 PMC9205785

[8] Wang J, Conlon D, Rivellese F, et al. Synovial inflammatory pathways characterize anti-TNF-responsive rheumatoid arthritis patients. Arthritis Rheumatol. 2022;74(12):1916–27. 10.1002/art.42295. 35854416 10.1002/art.42295

[9] Cuppen BV, Welsing PM, Sprengers JJ, et al. Personalized biological treatment for rheumatoid arthritis: a systematic review with a focus on clinical applicability. Rheumatology (Oxford). 2016;55(5):826–39. 10.1093/rheumatology/kev421. 26715775 10.1093/rheumatology/kev421

[10] Aletaha D. Precision medicine and management of rheumatoid arthritis. J Autoimmun. 2020;110:102405. 10.1016/j.jaut.2020.102405. 32276742 10.1016/j.jaut.2020.102405

[11] European Union (2022) EMA confirms measures to minimise risk of serious side effects with Janus Kinase inhibitors for chronic inflammatory disorders. EMA/860610/2022.

[12] Ytterberg SR, Bhatt DL, Mikuls TR, et al. Cardiovascular and cancer risk with tofacitinib in rheumatoid arthritis. N Engl J Med. 2022;386:316–26. 10.1056/NEJMoa2109927. 35081280 10.1056/NEJMoa2109927

[13] Salinas CA, Louder A, Polinski J, et al. Evaluation of VTE, MACE, and serious infections among patients with RA treated with Baricitinib compared to TNFi: a multi-database study of patients in routine care using disease registries and claims databases. Rheumatol Ther. 2023;10:201–23. 10.1007/s40744-022-00505-1. 36371760 10.1007/s40744-022-00505-1 PMC9660195

[14] Smolen JS, Landewé RBM, Bergstra SA, et al. EULAR recommendations for the management of rheumatoid arthritis with synthetic and biological disease-modifying antirheumatic drugs: 2022 update. Ann Rheum Dis. 2023;82(1):3–18. 10.1136/ard-2022-223356. 36357155 10.1136/ard-2022-223356

[15] Caporali R, Germinario S, Kacsándi D, et al. Start RA treatment-Biologics or JAK-inhibitors? Autoimmun Rev. 2023;25:103429. 10.1016/j.autrev.2023.103429. 10.1016/j.autrev.2023.103429 37634678

[16] Taylor PC, Keystone EC, van der Heijde D, et al. Baricitinib versus placebo or adalimumab in rheumatoid arthritis. N Engl J Med. 2017;376(7):652–62. 10.1056/NEJMoa1608345. 28199814 10.1056/NEJMoa1608345

[17] Fleischmann R, Pangan AL, Song IH, et al. Upadacitinib versus placebo or adalimumab in patients with rheumatoid arthritis and an inadequate response to methotrexate: results of a phase III, double-blind, randomized controlled trial. Arthritis Rheumatol. 2019;71(11):1788–800. 10.1002/art.41032. (**Epub 2019 Aug 28**). 31287230 10.1002/art.41032

[18] Combe B, Kivitz A, Tanaka Y, et al. Filgotinib versus placebo or adalimumab in patients with rheumatoid arthritis and inadequate response to methotrexate: a phase III randomised clinical trial. Ann Rheum Dis. 2021;80(7):848–58. 10.1136/annrheumdis-2020-219214. 33504485 10.1136/annrheumdis-2020-219214 PMC8237199

[19] Aletaha D, Neogi T, Silman AJ, et al. 2010 rheumatoid arthritis classification criteria: an American College of Rheumatology/European League Against Rheumatism collaborative initiative. Ann Rheum Dis. 2010;69(9):1580–8. 10.1136/ard.2010.138461. 20699241 10.1136/ard.2010.138461

[20] Toth L, Juhasz MF, Szabo L, et al. Janus Kinase inhibitors improve disease activity and patient-reported outcomes in rheumatoid arthritis: a systematic review and meta-analysis of 24,135 patients. Int J Mol Sci. 2022;23(3):1246. 10.3390/ijms23031246. 35163173 10.3390/ijms23031246 PMC8836107

[21] McLornan DP, Pope JE, Gotlib J, et al. Current and future status of JAK inhibitors. Lancet. 2021;398:803–16. 10.1016/S0140-6736(21)00438-4. 34454676 10.1016/S0140-6736(21)00438-4

[22] Khosrow-Khavar F, Kim SC, Lee H, et al. Tofacitinib and risk of cardiovascular outcomes: results from the safety of tofacitinib in routine care patients with rheumatoid arthritis (STAR-RA) study. Ann Rheum Dis. 2022;81:798–804. 10.1136/annrheumdis-2021-221915. 35027405 10.1136/annrheumdis-2021-221915 PMC9117457

[23] Fang YF, Liu JR, Chang SH, et al. Comparative safety of Janus Kinase inhibitors and tumor necrosis factor inhibitors in patients undergoing treatment for rheumatoid arthritis. Int J Rheum Dis. 2022;25:1254–62. 10.1111/1756-185X.14414. 35923107 10.1111/1756-185X.14414

[24] Kremer JM, Bingham CO, Cappelli LC, et al. Postapproval comparative safety study of tofacitinib and biological disease-modifying antirheumatic drugs: 5-year results from a United States-based rheumatoid arthritis registry. ACR Open Rheumatol. 2021;3:173–84. 10.1002/acr2.11232. 33570260 10.1002/acr2.11232 PMC7966883

[25] Cho SK, Kim H, Song YJ, et al. Comparative effectiveness of JAK inhibitors and biologic disease-modifying antirheumatic drugs in patients with rheumatoid arthritis. Korean J Intern Med. 2023;38(4):546–56. 10.3904/kjim.2022.369. 37334513 10.3904/kjim.2022.369 PMC10338257

[26] Mok CC, So H, Yim CW, et al. Safety of the JAK and TNF inhibitors in rheumatoid arthritis: real world data from the Hong Kong biologics registry. Rheumatology (Oxford). 2023;2:kead198. 10.1093/rheumatology/kead198. 10.1093/rheumatology/kead198 37129549

[27] Baldi C, Berlengiero V, Falsetti P, et al. Baricitinib retention rate: ‘real-life’ data from a monocentric cohort of patients affected by rheumatoid arthritis. Front Med. 2023;10:1176613. 10.3389/fmed.2023.1176613. 10.3389/fmed.2023.1176613 PMC10336222 37448804

[28] Singh JA. The emerging safety profile of JAK inhibitors in rheumatic diseases. BioDrugs. 2023;37(5):625–35. 10.1007/s40259-023-00612-7. 37351790 10.1007/s40259-023-00612-7

[29] XELJANZ (tofacitinib). https:// www. acces sdata. fda. gov/ drugsatfda_ docs/ label/ 2021/ 20321 4s028 ,20824 6s013 ,21308 2s003 lbl. pdf. In. Silver Spring, MD: U.S. Food and Drug Administration; 2021.

[30] Bechman K, Subesinghe S, Norton S, et al. A systematic review and meta-analysis of infection risk with small molecule JAK inhibitors in rheumatoid arthritis. Rheumatology. 2019;58(10):1755–66. 10.1093/rheumatology/kez087. 30982883 10.1093/rheumatology/kez087

[31] Gialouri CG, Moustafa S, Thomas K, et al. Herpes zoster in patients with inflammatory arthritides or ulcerative colitis treated with tofacitinib, baricitinib or upadacitinib: a systematic review of clinical trials and realworld studies. Rheumatol Int. 2023;43(3):421–35. 10.1007/s00296-022-05270-6. 36635577 10.1007/s00296-022-05270-6 PMC9968274

[32] Mehta B, Pedro S, Ozen G, et al. Serious infection risk in rheumatoid arthritis compared with non-inflammatory rheumatic and musculoskeletal diseases: a US national cohort study. RMD Open. 2019;5(1):e000935. 10.1136/rmdopen-2019-000935. 31245055 10.1136/rmdopen-2019-000935 PMC6560658

